# Taphonomic patterns of a WWI Alpine mass grave: insights from the Italian front

**DOI:** 10.1038/s41598-025-32171-y

**Published:** 2025-12-22

**Authors:** Wiktoria Baranowska, Mauro Gobbi, Stefano Vanin, Franco Nicolis, Daniel Gaudio

**Affiliations:** 1 Department of Archaeology, University of Durham, Durham, UK; 2 Climate and Ecology Unit, Research and Museum Collections Office, MUSE-Science Museum, Trento, Italy; 3 Department of Earth, Environmental and Life Sciences-DISTAV, University of Genoa, Genoa, Italy; 4 Archaeological Heritage Office of the Autonomous Province of Trento, Trento, Italy

**Keywords:** Alpine environment, Forensic taphonomy, Mass grave, Pink teeth, Reddish staining, World War I, Ecology, Ecology, Evolution

## Abstract

Understanding taphonomic patterns on skeletal remains, along with associated entomological evidence, remains a critical challenge in forensic and archaeological investigations. This study examines the specific impact of an Alpine environment on a WWI mass grave of 12 Austro-Hungarian soldiers (Cima Cady, Italy). Alongside general poor preservation caused by acidic soil erosion, a distinctive reddish staining affected over 80% of the skeletal elements. This was linked to the invasive root system of *Juniperus sp.*, a plant species typical of Alpine zones, which had penetrated the grave and bone cavities. Additional unusual deterioration patterns were observed on tarsal elements that had been in contact with leather boots. Entomological evidence included the presence of *Pterostichus multipunctatus* and puparia of *Protophormia terraenovae*, known to mainly colonise exposed remains. The combined taphonomic and entomological findings provide important insights into the postmortem history of the remains and the environmental factors influencing their preservation. Collectively, these results support historical records and testimonies of the burial dynamics from over a century ago, and highlight the broader forensic potential of such evidence in reconstructing mass grave scenarios, including the contemporary investigations of human rights violations or war crimes.

**Supplementary Information:**

The online version contains supplementary material available at 10.1038/s41598-025-32171-y.

## Introduction

Taphonomy is a well-established scientific field and includes forensic anthropology and human bioarchaeology among its applications. However, research focusing on human remains buried in Alpine environments, especially in wartime context, remains limited^1,2^.

This study uncovers the taphonomic evidence that has aided in the reconstruction of past events from World War One (henceforth WWI, worldwide: 1914–1918, Italian Front: 1915–1918), corroborates testimony taken from accounts older than 100 years, and provides a taphonomic pattern of the Alpine environment, characterised by lush meadows, which may provide valuable insights for the interpretation of evidence in both Alpine archaeological contexts and contemporary forensic investigations. This analysis, focusing on human remains from a WWI mass grave discovered in the Italian Alps, encompasses aspects such as the preservation of the skeletal remains, botany, and entomology. More specifically, it will detail the preservation of the skeletal assemblage, the peculiar and previously undocumented staining patterns observed on some of the skeletal elements, and the significance of the entomological evidence recovered from human remains over 100 years old. Together, these unusual findings offer valuable insights into the sequence of events leading to the victims’ burial.

### Context of the case

**Fig. 1:**
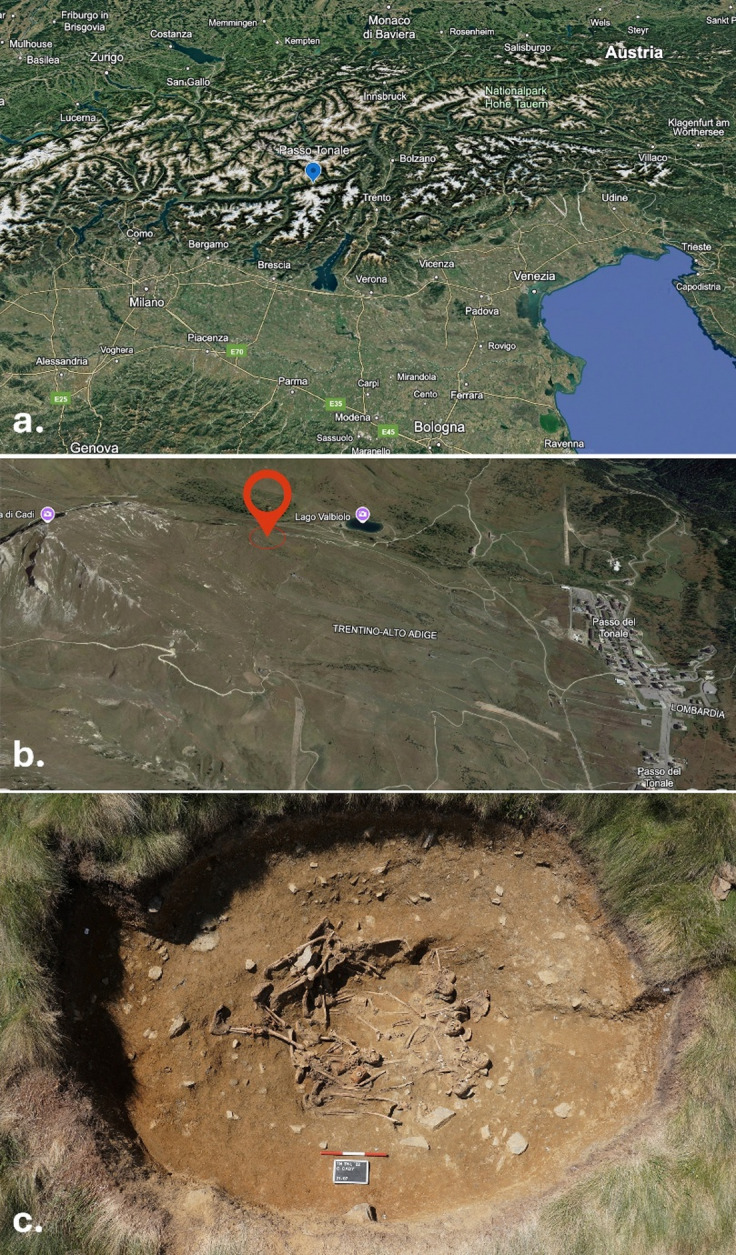
Location of the mass grave — (**a**) Tonale Pass; (**b**) the area where the mass grave was found in Cima Cady^3^; (**c**) the photograph of the mass grave itself (Image: Archive of the Archaeological Heritage Office, Trento; photo by Marco Redaelli, SAP^4^.

The skeletal remains, upon which this study is based, were discovered within a mass grave located 2309 m above sea level, on the southern slope of Cima Cady in the municipality of Vermiglio (Southern Rhaetian Alps, Italy), as illustrated in Fig. 1. The mass grave was situated inside the crater formed by an explosion of a grenade during WWI. The human remains in the mass grave belonged to Austro-Hungarian soldiers who fought during Operation Avalanche from 12 to 13 June 1918, as supported by the presence of military equipment, personal items associated with the remains, as well as the military documentation and notes still preserved by a grandson of a soldier who served in Cima Cady. Operation Avalanche was an Austro-Hungarian offensive launched as part of the broader June 1918 campaign, commonly referred to as the Battle of the Solstice (*Zweite Piaveschlacht*). The operation aimed to break through the Italian front in the Alpine sector and advance into the lowlands, seeking to surprise and overwhelm Italian forces. These kinds of military actions underscore the critical role of the Alpine environment and the formidable logistical challenges inherent to mountain warfare. The documentary research that led to the discovery of the mass grave was carried out by the descendant of the Italian soldier who had fought in the Tonale area. It involved comparing the soldier’s front-line diary with the described location, where he mentioned a mass grave reportedly containing ‘enemy’ soldiers. Further comparison also considered the current *warscape* of Cima Cady, where trenches, strongpoints, and craters from grenade explosions are still visible today. This process led to the identification of a large shell crater that appeared to match the one described in the diary. Considering the details described in the diary, the soldiers buried there were likely interred by the Italian army as they hastily withdrew from the battlefield.

The location, excavation, recovery and analysis of the remains were coordinated by the Archaeological Heritage Office of the Autonomous Province of Trento (Italy) in collaboration with Durham University (UK), MUSE-Science Museum of Trento (Italy) and the Italian Ministry of Defence, which constitutes the responsible authority for this type of investigation in Italy. Fieldwork was carried out by SAP Società Archeologica under the scientific direction of one of the co-authors. Twelve individuals — adult males aged approximately 18 to 35 — were found within the mass grave. Of these, three showed high-velocity projectile trauma, two presented blast/projectile trauma, and the remaining individuals exhibited no visible trauma. Their skeletal remains partially overlapped and laid in various positions, such as prone, supine or lateral, facing different directions. In 2024, the soldiers were ceremonially interred with honour at Castel Dante in Rovereto, Italy, alongside other Austro-Hungarian soldiers who fell on WWI battlefields in the Alps. Additionally, several personal and military items were found among the human remains — those included gas masks, leather fragments, identification plates in brass (some with shreds of written paper), crampons, buttons, cartridges, small glass containers for ointment, pencils, an eraser, and iron buckles. The items are currently stored and preserved at the Restoration Laboratory of the Archaeological Heritage Office of Trento, Italy.

### Taphonomy of mass graves

Taphonomic data can aid the investigation of mass graves, for example, by corroborating witness testimony^5^ or estimating the time elapsed between death and the burial of an individual based on entomological evidence^6–9^. However, the taphonomic environment of the burial site can also complicate aspects of skeletal analysis. For instance, Gordon and Buikstra (1981)^10^ demonstrated that the lower the pH of the soil at the burial site, the greater the destruction of the skeletal material, a problematic effect in both bioarchaeological and forensic contexts^11^. Contact between the remains of different individuals, as well as contact with the surrounding military items, can also highly affect the state of preservation of the human remains. An example of this is the ‘feather edge effect’ often observed in the mass grave setting: bodies located around the edge of the grave decompose faster than those situated in the centre of the ‘body mass’^12^. This phenomenon can obscure the establishment of context and complicate the reconstruction and sequencing of events critical to the investigation^13^. Previous studies have not, however, included Alpine contexts where additional environmental factors such as temperature extremes, specific vegetation and geomorphology in the mass grave can present further challenges to skeletal analysis.

### Environmental context of the Cima Cady site

The location of the study’s mass grave — Alpine zone of Cima Cady — is currently subjected to varying temperatures: on the valley floors, January temperatures between − 5 and 4 °C are common, rising to 15 and 24 °C in June. At higher altitudes, even higher extremes can be observed with colder winters and hotter summers^14^.

The Alpine zone, one of the five climatic zones/belts of the Alps, can be characterised by lush meadows, with some areas eroded by glaciation^14^. Meadows typically feature herbaceous plants like Poaceae and Cyperaceae, and shrubs of *Salix*, *Juniperus* and *Vaccinium*^15^. The soil in the Alps is characterised by a highly diverse geological substratum, including calcareous sedimentary rocks such as limestone and dolomite, as well as metamorphic rocks, slate, plutonic and volcanic rocks, and a range of gravitational, fluvial, aeolian, and glacial deposits with varying textures^15^. Both the Alpine vegetation and the geological elements are recognised as taphonomic factors that can contribute to mechanical disruption of the remains, the chemical alterations of skeletal elements (e.g. root-induced damage or bone dissolution via altered soil pH ^10^), and even cause confusion at the analysis stage, where root marks might be misinterpreted as skeletal trauma^16^.

At higher elevations of the Alpine zone, pseudogleyification (waterlogging of soil due to poor drainage), and element leaching can be observed due to higher precipitation, snowmelt and slope run-off. As a result of leaching and acidification processes, soil pH values decrease with an increase in altitude^17^. This effect, additionally amplified by the presence of Alpine vegetation, can lead to the greater destruction of the skeletal elements found in this setting.

According to the archaeological report of the excavation^4^, the exposed stratigraphy comprised slope deposits and undifferentiated glacial sediments, overlain by colluvial layers (US 4 and US 3) in which a weakly developed soil (Entisol) has formed. The crater (US 5), resulting from the detonation of a large-calibre shell, was subsequently repurposed as a collective burial site for Austro-Hungarian soldiers. The overlying deposit (US 2) derived from both intentional backfilling – likely undertaken by Italian troops in June 1918 – and natural downslope sedimentation. Vegetation in the uppermost layer (US 1) appeared particularly vigorous, probably due to enhanced soil moisture within the crater. The vegetation present on the mass grave site was typical of the Alpine zone^15^, consisting mainly of grass (Poaceae, Fescues) with heather, bluebells, Alpine anemones, blueberry, arnica, and gymnadenia. Rhododendrons and juniper bushes were also covering the grave site. Additionally, there were some sparse plants, including common larch, two-coloured widow, the alder and red picea. Edelweiss was visible above the site^4^. The crater was of elliptical shape, with overall diameter measuring approximately 6 m; it also featured a deeper, central area described as approximately 2.70 m in diameter. It consisted mostly of a superficial soil layer, a filling deposit measuring about 0.70–0.80 m in thickness (the layer including all skeletal remains), and a sterile soil layer on the bottom of the crater with a maximum thickness of 0.35 m^4^). Total depth of the crater was equal approximately 1.5 m. The pH of the soil adjacent to the human remains was measured to be between 3.83 (individual S11, deepest burial) and 4.91 (individual S10, mid-to-upper burial position). The pH of the soil recovered from inside of the boot of the individual S6 was slightly less acidic, with a value of about 5.20.

Generally, each burial site has its specific taphonomic character, with extrinsic factors such as soil pH, flora or fauna^18^ leaving an overall distinct signature on the human remains. The specific burial conditions in Alpine environments have received limited research and, therefore, are not well known. In light of these complex environmental factors and the scarcity of studies linking Alpine zone meadows to the analysis of human remains, this study aims to characterise the distinctive taphonomic pattern produced under these specific meadow conditions, and to determine whether the combination of taphonomic factors can help reconstruct the events surrounding the deaths of the Austro-Hungarian soldiers found in the mass grave at Cima Cady. Several of the taphonomic alterations examined — such as pink bone staining or vertical erosion of the tarsals, to the best of the author’s knowledge, have received little to no attention in previous research and could have implications for both forensic casework and bioarchaeological investigations.

## Methods

The human remains were recovered from the mass grave using archaeological methods, including stratigraphic excavation. Twelve individuals were subjected to skeletal and taphonomic analysis. Each subject was labelled with ‘S’ (i.e., ‘Subject’) and the corresponding number, for example, S1. Based on the position of the bodies in the grave and the skeletal analysis, it was established that skeletal elements originally labelled separately as S9 and S14 most likely belong to the same individual; similar conclusions were drawn for S8 and S13, as well as for S7 and S10. Accordingly, the analysis reported in this study involved 12 individuals.

### Skeletal preservation

Preservation of the skeletal elements was assessed using a modified Bello et al.^19^ recording system. The skeletal indices were modified to improve their functionality for the purpose of this study. In this research, the Anatomical Preservation Index (API) evaluates the quantity of osseous material present^19^. The Qualitative Bone Index (QBI) assesses the state of cortical surface preservation^19^, and it is scored only on the available bone parts, ignoring missing fragments. Bone Representation Index (BRI) measures the ratio between the actual number of recovered bones present and the total expected number of bones, therefore offering a measure of skeletal completeness. Several aspects of Bello et al. ^19^ were modified, as explained further, to clarify some aspects of the approach adopted. Each bone with an API value higher than 1 was counted as present. Vertebrae, ribs, and bones of the hands and feet were counted as individual bones, whereas Bello et al.‘s system grouped them as single units. The sacrum was counted as a single element, while the sternum, manubrium and xiphoid process were counted as three separate bones. According to Bello et al.^19^ definition, a bone was only considered well preserved if more than 50% of the bone was present (API > 3) or it had more than 50% of sound cortical surface (QBI > 3). The skeletal remains were only considered well preserved if more than 50% of the scored bones had an API higher than 3.

**Table 1:** A skeletal fragmentation scoring system developed and used for the purpose of this study.

Skeletal fragmentation scoring system	
Score	Description
1	Absent — no bone present
2	Very severely fragmented — bone crushed into a high amount of very small fragments (less than 5 mm)
3	Severely fragmented — bone broken into many small fragments (less than 1 cm in size).
4	Fragmented — bone fragmented into several fragments;
5	Slightly fragmented — bone intact with a missing portion or fragmented into less than 3 big fragments
6	Intact — complete, intact bone present

A new fragmentation scoring system, aligned with the 6-class Bello et al.^19^ preservation scoring system, was developed for this study. Accordingly, bone fragmentation was scored as outlined in Table 1. Skeletal indices, as well as fragmentation score, were not assigned to cartilages, auditory ossicles, sesamoid bones, or any supernumerary or unidentified/not-sided bones.

Average API, QBI and Fragmentation (counting all bones and then counting only present bones) were calculated for each individual and the whole assemblage. These were calculated for all skeletal elements contained within the soil, and, separately, for foot bones contained within the boots.

In addition, the information regarding the position of human remains in the grave was retrieved from the original archaeological site report^19^.

Kruskal-Wallis statistics test was run to determine whether there is a significant statistical difference between preservation indices of human remains found at different burial depths within the mass grave.

Moreover, other basic summary statistics, such as average and standard deviation calculations or plotting of the data using Microsoft Excel, were implemented to summarise the data retrieved from the analysis of skeletal preservation.

### Taphonomic evidence

Taphonomic alterations such as root impressions, extrinsic erosion, or bone staining were documented using tailored skeletal recording forms and a Nikon D850 camera. The location and appearance of taphonomic changes were described, and potential causes were assigned. The measurements were taken of any alterations visible only in a particular place on the skeleton. Colours of the bone staining were described.

The samples of the roots invading the human remains were collected, and the pH of the soil samples (total of 4 diluted soil samples) taken from the soil adjacent to the human remains and recovered from the boots was tested using HI-991,300 Waterproof pH, EC, TDS and Temperature Meter with Advanced Features.

### Entomological analysis

Entomological samples were collected, the external auditory meatus in subject S1 from the inner area of the cranium, as well as from the inner area of the cranium in S4 and S6. The samples were then cleaned following the protocol of Pradelli et al.^20^ and analysed using a Leica M60 stereomicroscope and identified by entomological keys, specific literature^21,22^ and comparison with reference collections.

### Ethics statement

All human remains analysed in this study were handled with utmost respect and in accordance with ethical guidelines for archaeological and forensic research, and Italian law. The investigation was carried out under the supervision of the relevant local authorities. No invasive procedures were performed beyond what was necessary for scientific analysis, and all efforts were made to preserve the dignity of the individuals.

## Results

### Skeletal preservation

Table 2 summarises the preservation (API – Anatomical Preservation Index, QBI – Qualitative Bone Index), fragmentation (F: for elements with API value higher than 1), and Bone Representation Index (BRI) of the human remain from Cima Cady mass grave. Scores for all indices were calculated for each individual as well as the whole assemblage contained within the soil environment. The indices for the foot bones (tarsals and metatarsals) contained within boots were omitted from these calculations as they were preserved by the exclusive environment of the boots and, consequently, were calculated separately.

**Table 2:**
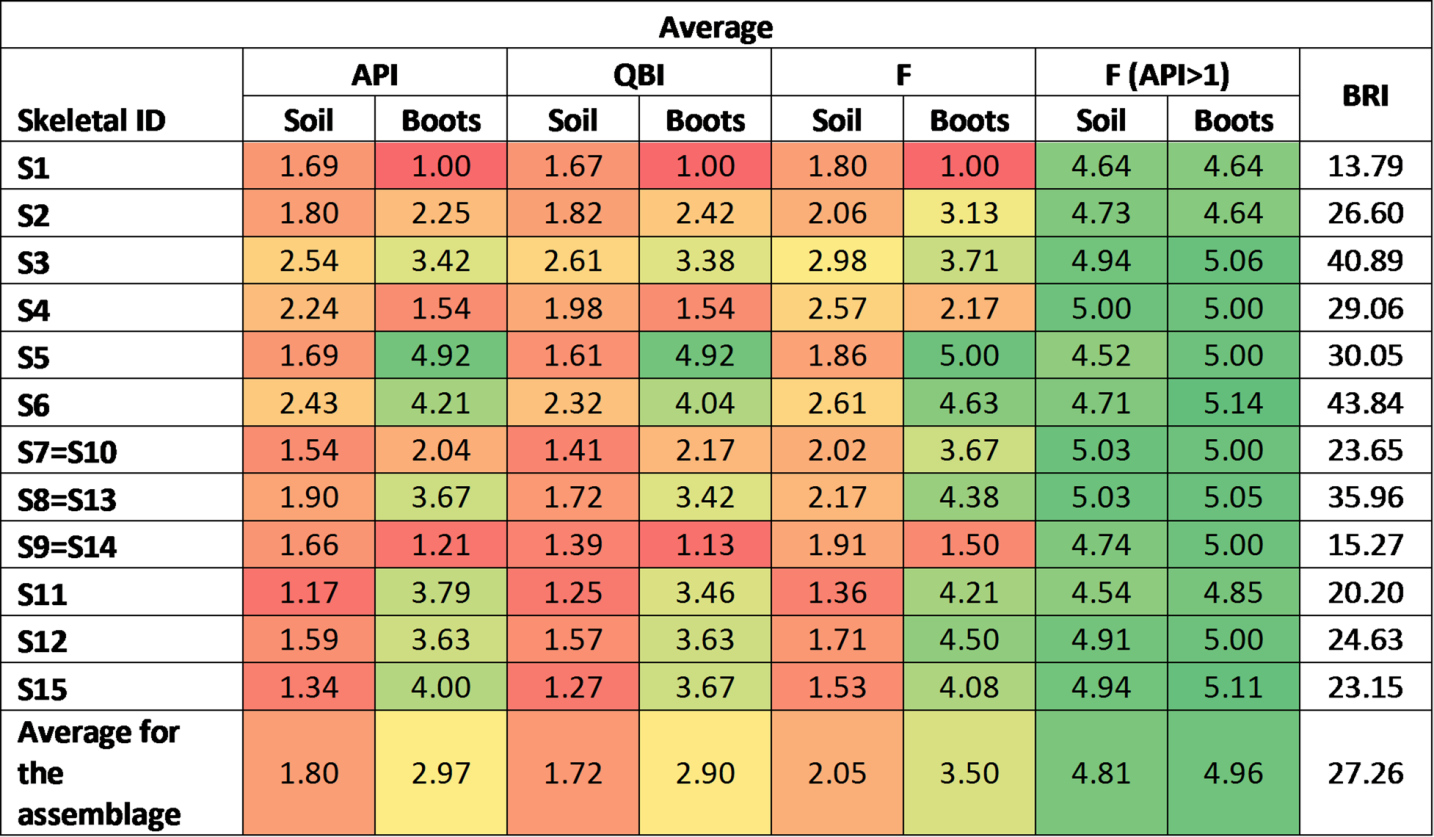
The preservation and fragmentation scores for each individual and the whole skeletal assemblage contained within the soil environment. The colour scale in the table is a visual aid, with green colour corresponding to very good preservation, and red colour corresponding to poor preservation or not preserved remains. As mentioned in the methods section, S7 and S10, S8 and S13, as well as S9 and S14, were originally marked as 6 separate individuals; however, in further stages of excavation, as well as based on the skeletal analysis, they were determined to belong to the same individual (S7 = S10, S8 = S13 and S9 = S14).

The percentage of well-preserved bones in relation to the total bones to be recorded for each skeletal remains was also calculated. The results of this analysis show that none of the skeletal remains had more than 50% of bones well preserved, based on either the API or QBI. Therefore, according to the criteria outlined by Bello et al.^19^, none of the skeletal assemblage was considered well preserved.

**Fig. 2:**
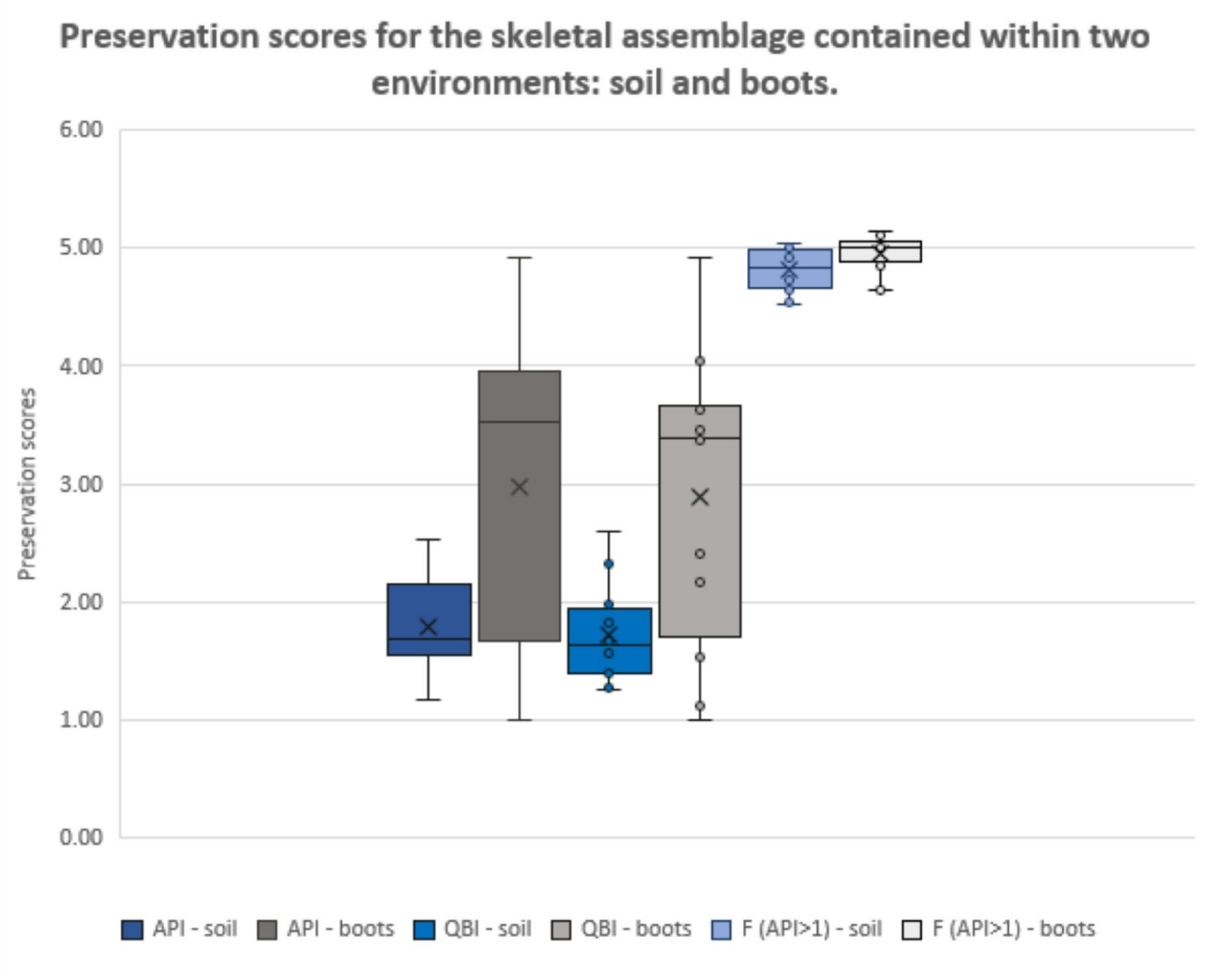
Comparison of preservation indices (API and QBI) and fragmentation (for elements with API > 1) for the skeletal assemblage contained within the soil and the one contained within the boots (boots associated with S3, S5, S6, S8 = S13, S11, S12, S15). The box represents the interquartile range (IQR) from the first quartile (Q1) to the third quartile (Q3), with a line at the median (Q2). Whiskers extend to the minimum and maximum values within 1.5 × IQR. Points outside this range are considered outliers and are pictured as circles, while X represents the mean.

Different preservation patterns were observed for the skeletal elements from two settings — soil and boots. These provided distinct environments for the human remains, each leaving a unique imprint (Fig. 2), with skeletal elements contained within boots being generally preserved better than elements contained within the soil. Some human remains (S1, S4, S9 = S14) can be observed as distinct outliers in this comparison (with skeletal elements preserved better in soil rather than boots) – this occurrence can be explained for these individuals by the lack (S1) or only limited number (S4, S9 = S14) of foot bones being recovered directly from the soil – in all these cases no boots were found associated with the human remains.

To evaluate the influence of burial depth on skeletal preservation within the mass grave, the remains were first assigned to one of four depth categories — low (S11, S12, S8 = S13), mid-low (S3, S15), mid-high (S2, S4, S5, S7 = S10), and high (S1, S6, S9 = S14) — based on their depositions in the mass grave (see Supplementary Figure S1 for a diagram of position of the human remains in the mass grave).

As mentioned in the Introduction under 'Environmental context of the Cima Cady site’, the pH of the soil adjacent to the human remains lower down in the grave (S11) was lower than in the soil adjacent to the remains higher in the column (S10). Based on this observation and due to the well-studied link between soil pH and preservation of the skeletal remains, it was hypothesised that individuals interred at greater depths might exhibit poorer preservation than those buried nearer the surface. Skeletal preservation was quantified using established preservation scores, and the null hypothesis (H₀) stated that no significant differences in preservation scores exist among the four burial-depth groups. The alternative hypothesis (H₁) asserted that burial depth correlates with preservation quality.

A statistical comparison (see Supplementary Table S2) of preservation scores across the depth categories (α = 0.05) yielded a p-value > 0.05, indicating no statistically significant differences among groups. Consequently, H₀ could not be rejected, suggesting that, within this mass-grave context, burial depth alone does not predict preservation state.

### Taphonomic alterations

**Fig. 3:**
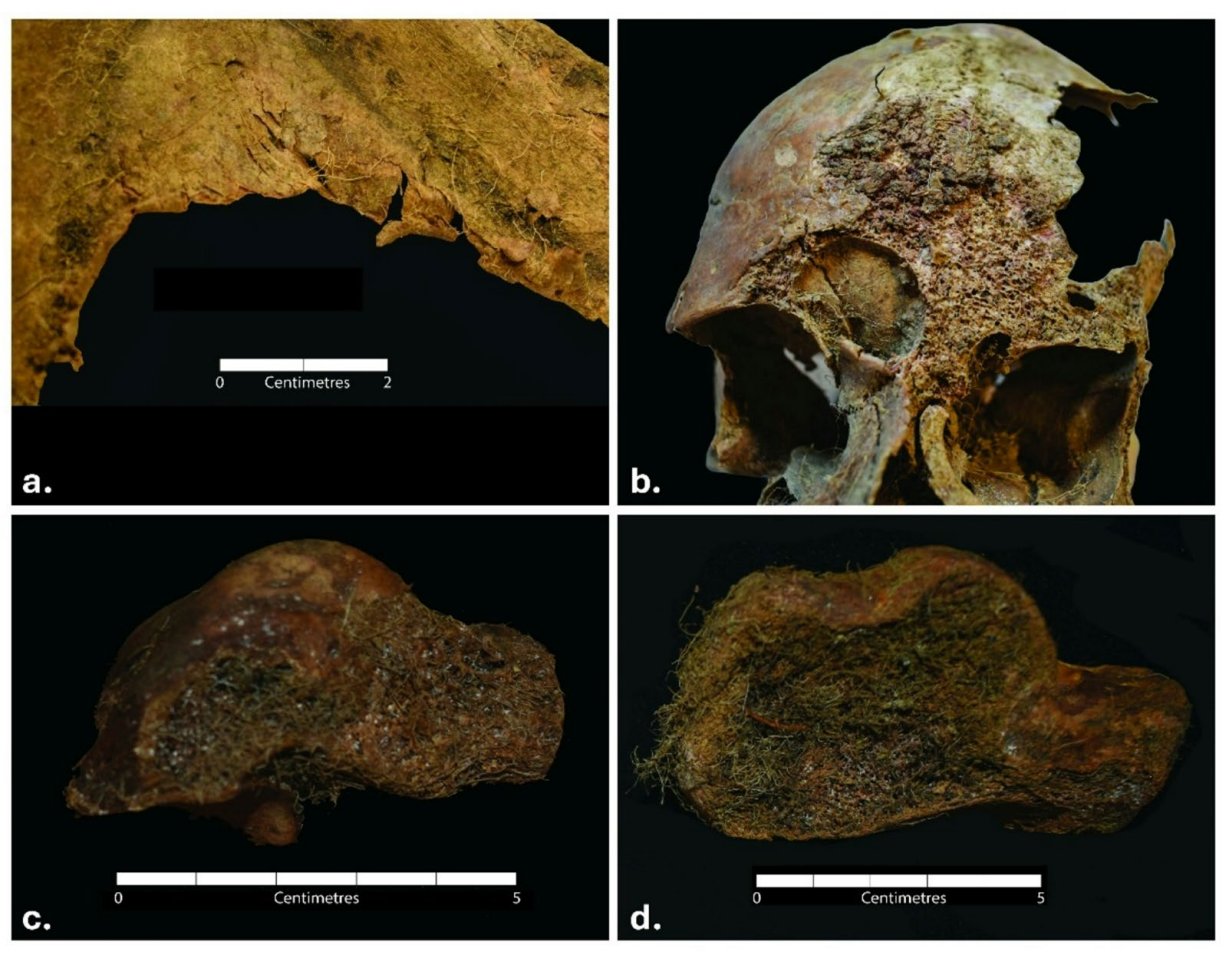
Erosion of the skeletal elements in the individuals recovered from Cima Cady. (**a**) Thinning of the bone edge in the scapular blade of S6; (**b**) erosion of the frontal bone of S6; (**c**) vertical loss of cortex on the lateral side of calcaneus of S15, and b. vertical loss of cortex on the lateral side of the talus of S6.

Extrinsic erosion of the skeletal material was observed throughout the whole assemblage buried within the soil of the mass grave. The cortical surface of most bones was severely damaged, with the periosteal surface often completely missing and the edges of the flat bones largely thinned (Fig. 3, a. and b.). In most cases, the epiphyses of long bones were completely eroded — the severity of cortex damage led to exposure of trabeculae.

While the foot bones preserved within the boots of the individuals generally presented much better preservation than the skeletal elements contained within the soil, a peculiar pattern of erosion was observed. Where cortical bone was in contact with the boot leather, a severe erosion was visible, with the trabecular bone fully exposed. Figure 3 (c. and d.) shows an example of an unusual vertical loss of the lateral cortex surface resulting in a ‘slit off’ or ‘cleft-like separation’ appearance of the tarsals.

**Fig. 4:**
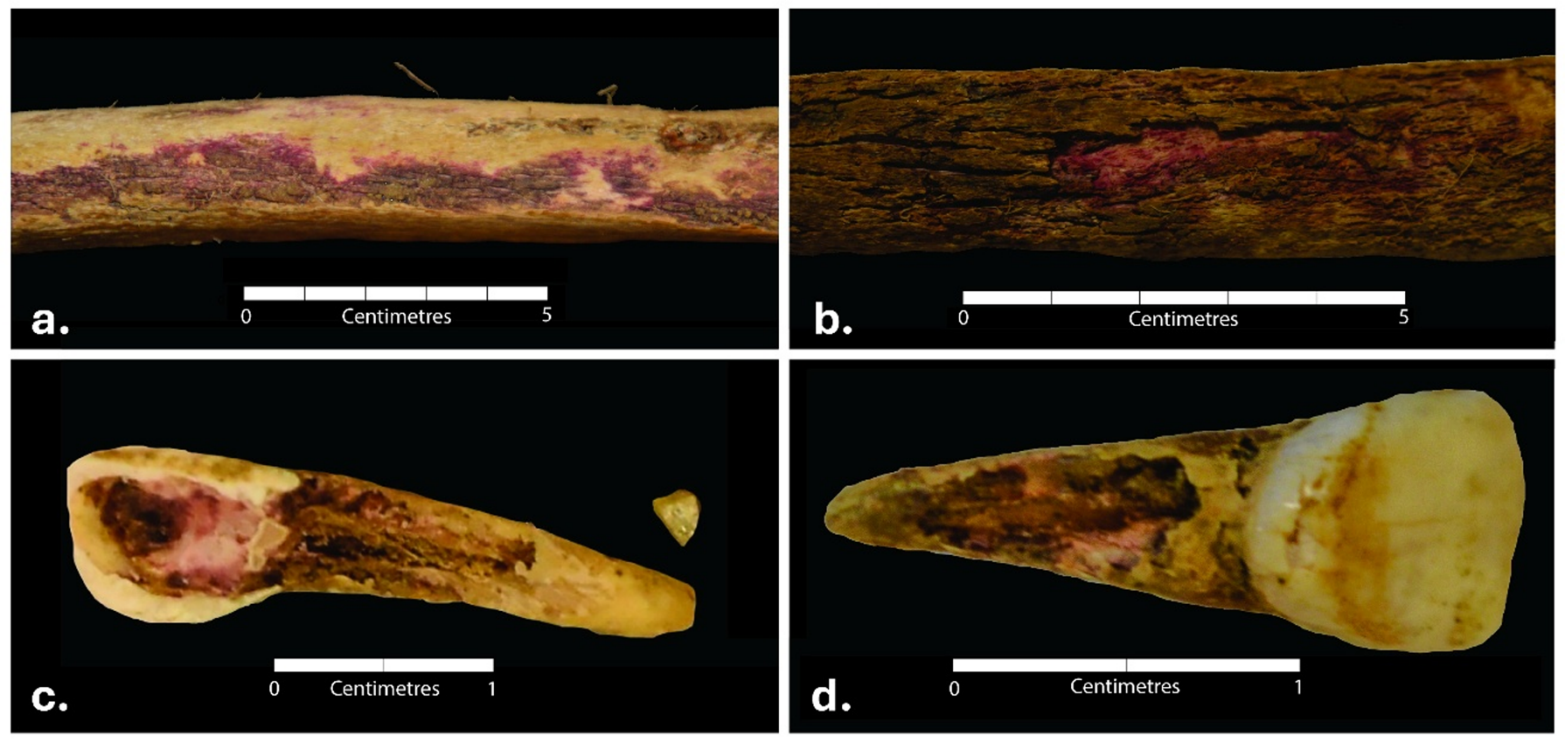
Examples of reddish staining present on the shafts of long bones of S1 (**a**) and S2 (**b**), and coronal dentine in the upper left canine of S4 (**c**), as well as root dentine in the 1st right upper incisor of S6 (**d**).

The most evident alteration present throughout the whole assemblage was the reddish staining of the skeletal material, present on almost every bone in the assemblage (minimum 80% of the skeletal elements), especially all long bones. This phenomenon was best visible on the skeletal elements with heavily eroded surfaces, as the most vibrant staining was accumulated on the layer of cortical bone under the outer periosteal surface of the bone cortex (Fig. 4, a. and b.). No staining was present on the top of the periosteal cortex; some evidence of staining was visible on the medullary cavity lining, and on the trabecular bone of the tarsals (where the cortical bone was completely eroded).

Reddish staining was also identified in the dentine layer of the teeth broken Post-Mortem (Fig. 4, c. and d.) — coronal dentine in S4 (upper left canine), root dentine in S6 (upper right first incisor).

Furthermore, other types of staining were found on the skeletal material, including green, orange, black and dark red staining as well as bleached discolouration of the bones (see Supplementary Table S3 for details).

### Additional taphonomic evidence: botanic elements and entomological evidence

**Fig. 5:**
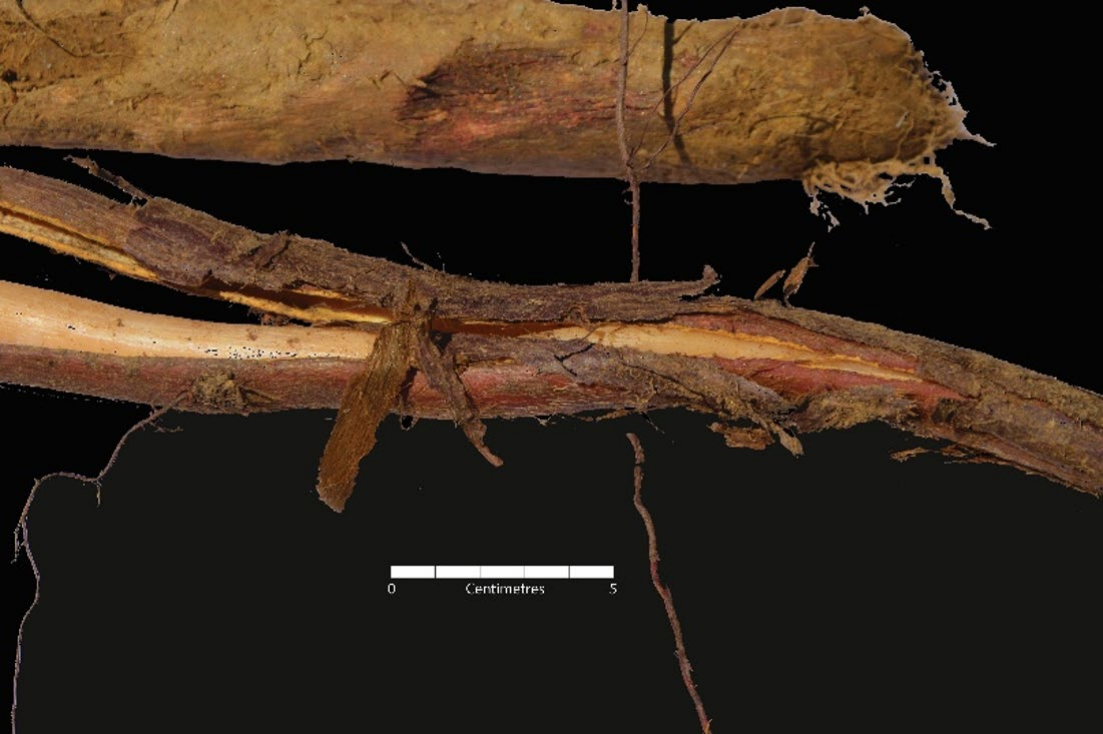
Juniper roots entwined with remains found within the mass grave.

Several roots were found within the mass grave, some of which were tangled around the skeletal elements and penetrated into them. The larger, reddish (‘wine-coloured’) roots recovered from the mass grave were identified as belonging to the *Juniper* species (Fig. 5). A link between these distinctive roots and the bone staining will be discussed further.

**Fig. 6:**
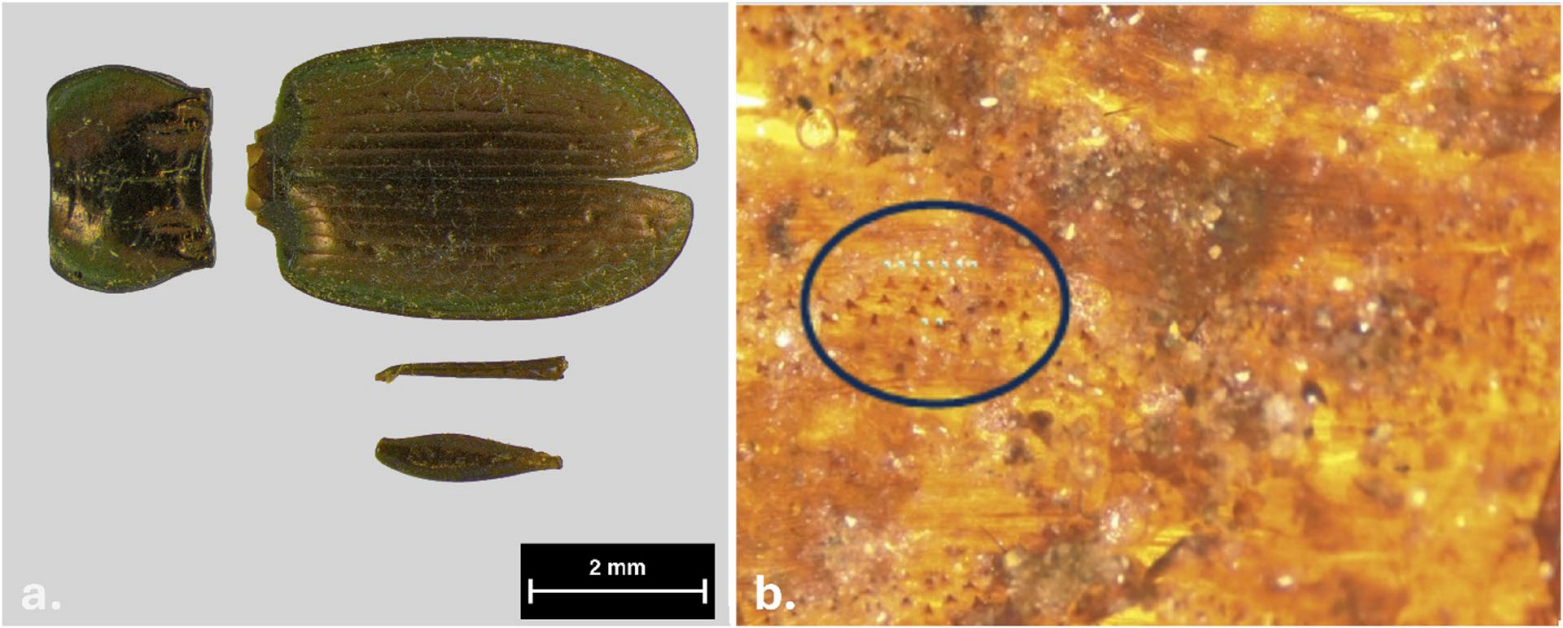
Entomological evidence: (a) *Pterostichus multipunctatus* associated with S1 (Photo by Francesco Simone Mensa/MUSE Archive); (b) the area where bifurcated spicules are present, a key diagnostic feature of the species *Protophormia terraenovae*.

The analysis of entomological samples revealed the presence of fragments belonging to the insect orders Coleoptera and Diptera, in particular some well-preserved exoskeleton parts of the ground–dwelling beetle: *Pterostichus multipunctatus* (Dejean, 1828) (Coleoptera: Carabidae) (Fig. 6). The fragments of this species were associated with the external auditory meatus of S1. *Pterostichus multipunctatus* is a ground-dwelling species.

Other entomological materials found, specifically on S1, S4, and S6, consisted of puparia fragments structurally attributable to flies (Diptera) of the family Calliphoridae. The examination of intersegmental spicules confirmed that all puparia belonged to a single species within the subfamily Chrysomyinae, specifically *Protophormia terraenovae* (Robineau-Desvoidy, 1830) (Fig. 6).

Although this study focuses on taphonomic aspects, the biological profiles, pathologies, and traumas of the individuals are provided in the Supplementary Information (Supplementary Table S4).

## Discussion

The taphonomic analysis of human remains recovered from the WWI mass grave at Cima Cady integrates skeletal preservation data with botanical and entomological evidence. This multidisciplinary approach offers valuable insights into the unusual taphonomic patterns characteristic of Alpine settings, with applications across a range of forensic and archaeological scenarios. The preservation of the skeletal remains in this study was very poor, with large portions of the skeletal elements missing and the cortical surface heavily eroded, resulting in low API and QBI score values. While the skeletal remains were largely weathered and incomplete, only minor fragmentation was observed, but it is likely that missing, fragmented elements were so severely eroded that they were not recovered or identified. The erosion of the skeletal material within the soil can be likely linked to the acidity of the soil at the burial site (situated at a high altitude of the Alpine zone), and the physical action of plant’s roots: Gordon and Buikstra (1981)^10^ proved that there is a strong relationship between the pH of the soil and the preservation of the skeletal remains. Even weak acidic solutions are known to have a destructive impact on inorganic bone matter^23^. This, together with the mechanical destructive action of the roots invading the human remains, could be the cause of very poor preservation of human remains at the Cima Cady site.

The bones contained within the boots were better preserved than those in the surrounding soil. The pH of the soil recovered from the interior of the boot (of individual S6) was, on average, higher (5.20) than in the surrounding soil, which could potentially explain the preservative property of the boot. As the human remains within the boot decomposed, the pH of the soil was potentially increased due to the release of ammonium ions^24^. It is possible that the boot served as a protective barrier against physical and biological agents (ice, plant roots, micro and macro fauna), which might explain why the pH of the soil within the boot did not return to its original state over the years. The boots would have also protected the bone from water moving through the soil, as water movement is necessary for ion exchanges between bone and the surrounding environment, including the leaching of calcium ions.

On the other hand, the pattern of vertical erosion (Fig. 3) was evident on the lateral side of the tali and calcanei that were in direct contact with the leather of the boot. To the best of the authors’ knowledge, this pattern of erosion has not been previously investigated, and no information about the erosive properties of the boot leather was encountered. Nonetheless, generally, the pH of the leather is reported to be between 4 and 7^25^, and, therefore, classified as acidic. Knowing that acidic soils erode bone^10,23^, a similar association could potentially be made between the acidic boot leather and erosion of the cortex on the tarsals, with the direct contact between the two likely intensifying the impact. Metatarsals were not affected, likely as there was more space between these skeletal elements and the leather, which over time got invaded by the layer of roots, separating these bones from the leather and protecting them from its impact. Although experimental research exploring the properties of leather and its impact on human bone should test this hypothesis, it can be said that the leather boot likely affected the cortical bone, contributing to the vertical loss observed on the lateral sides of the tali and calcanei.

The additional preservation-related factor tested in this study considered the correlation between the burial depth of the human remains and their preservation. Although deeper burial is generally associated with better preservation due to more stable environmental conditions, a slightly lower pH value (3.83) was recorded for soil around the deepest-placed individual (S11) in comparison to soil associated with remains located mid-high in the grave column (4.91), raising the possibility that localised acidity might develop at depth. Low pH is strongly linked to poorer bone preservation, and certain alpine soil processes can plausibly generate acidic microenvironments even within calcareous settings. Feng et al.^26^ showed that high Soil Organic Matter (SOM) can enhance water retention and promote the formation of organic acids, potentially lowering pH, but this can be compensated by the buffering effect of dust-derived CaCO₃. Similarly, Djukic et al.^27^ documented marked pH variability in alpine Histosols, ranging from highly acidic conditions under acidophilic vegetation to substantially more alkaline pH where basophil species dominate, indicating that distinct microenvironments can form across short spatial scales. However, the results of the Kruskal–Wallis test did not support this hypothesis. This outcome indicates that, in this specific Alpine mass grave context, the conventional taphonomic patterns appear consistent, indicating that the preservation of the remains is governed by a complex interplay of multiple factors^28^ – such as microenvironmental conditions, differential plant root intrusion, leather contact, and localized water movement – had a stronger influence on the state of the remains than the vertical position within the grave. Another limitation of the study is that pH measurements were not obtained directly from the grave matrix during excavation. Rather, they were taken from soil adjacent to the remains, which prevents a definitive assessment of whether pH decreased with depth within the grave. Nevertheless, the relationship was explored to ensure completeness of the analysis.

**Fig. 7:**
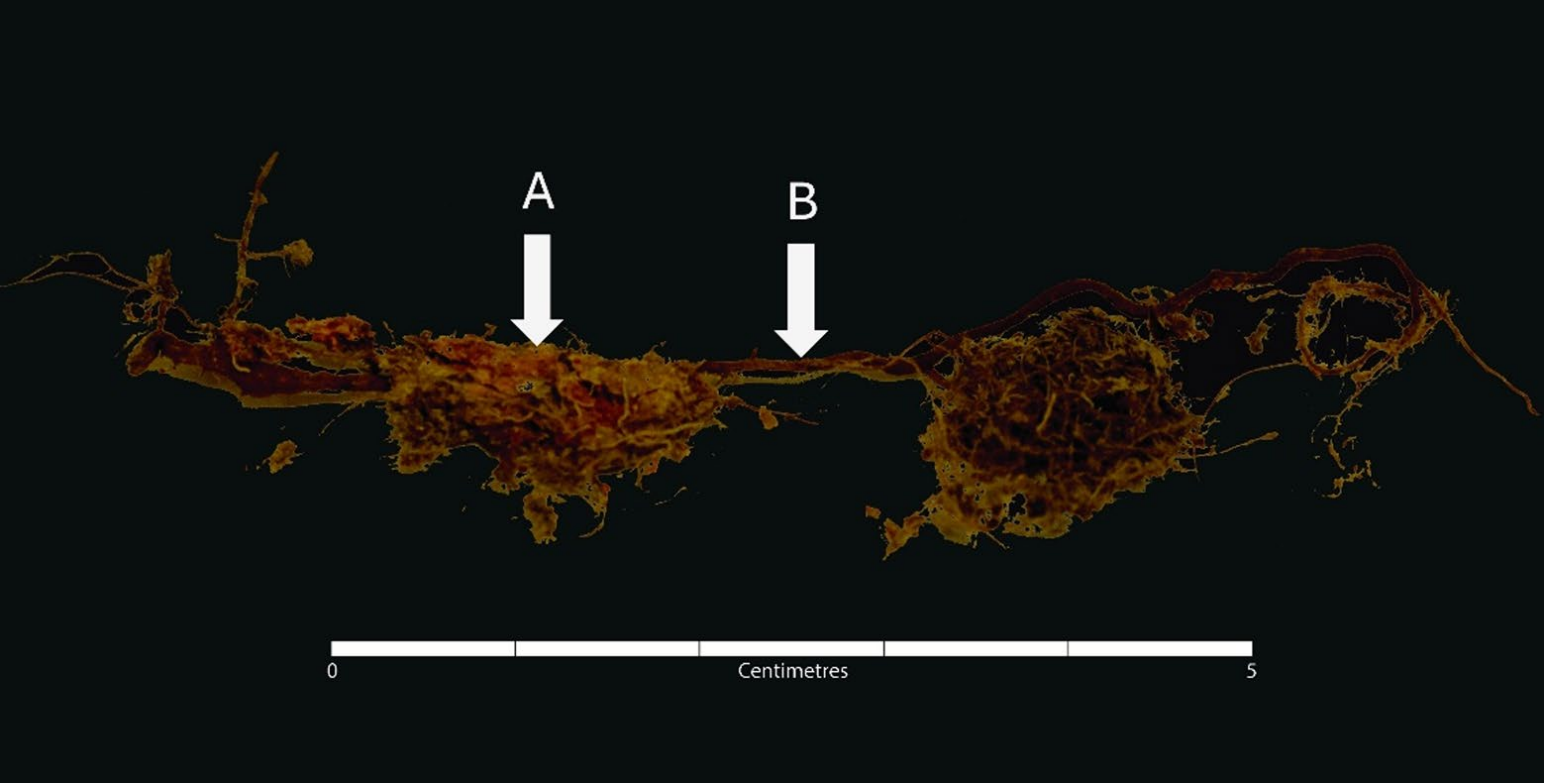
A close-up of wine-coloured Juniper root (B) attached to pink-stained, crumbling bone fragments (A) of S8 = S13.

The most peculiar taphonomic feature encountered during the analysis (minimum 80% of recovered skeletal material) was a reddish staining of bones and teeth. As the red-coloured Juniper roots creating tight layers were often adjacent to the areas of pink/purple staining on the skeletal material (Fig. 7), as well as were observed invading the foramina and stained medullary cavities, the link between the two occurrences was strongly supported.

Juniper belongs to the family of evergreen conifers^29^, and was the only conifer found at the site. Conifers, like other trees, are known to contain phlobaphenes^30^ (also referred to as reddish-purple pigments), which can impart a reddish pigment on organic materials with which they come into contact. Depending on the plant species, phlobaphenes consist of cyanidins, pelargonidins, or other polyphenolic polyesters. It is reported^30^ that these pigments are ‘sparingly’ soluble in water but also relatively unstable in mildly acidic solutions. Considering the acidic nature of the soil at the site, the pigments were likely released from the Juniper roots and, with the flow of water produced by the rain and ice/snow melting in the higher altitude zone of Cima Cady, the pigments travelled through the soil and accumulated on the skeletal remains.

Notably, the reddish staining was found only as a thin layer under the outermost layer of the cortical bone, but never on top of the periosteal surface, and rarely on trabecular bone. This could be explained by the properties of the bone tissues. The Juniper roots penetrated the medullary cavities, which likely caused the pigment to follow a similar path as India ink in Li et al.^31^ study: the ink was able to pass from the medullary cavity through the trabecular bone pores, Haversian canals and canaliculi of deeper layers of cortical bone. The outer surface of cortical bone, instead being not permeable^31,32^, consequently, likely prevented the passage of pigment, leading to its entrapment as a thin layer beneath the periosteal cortex. When the outer cortex was eroded by the soil acidity, the pigment potentially had an ‘easier route’ and simply accumulated on the top of the exposed deeper cortical surface. The dense structure preventing the absorption of big dye molecules might be an answer to why the staining was not adherent to the periosteal cortex.

The differential permeability may explain why intense reddish staining was also found in the dentine and lining of the root canal of the teeth broken post-mortem, but was not evident on the enamel. Dentine owes its high permeability to the presence of microscopic dentinal tubules surrounded by the collagen fibrils^33^. The diameter of the tubules decreases closer to the enamel/cementum. The enamel of the teeth does not contain tubules and is highly mineralised, therefore, it can be concluded that its permeability level is low^34,35^. Furthermore, dentine potentially allowed for the passage of the reddish pigment through its layers, while the enamel acted as a barrier for the staining flow, causing it to accumulate in the dentine layer. The higher intensity of coronal dentine staining in comparison to root dentine staining could also be explained by the higher permeability of coronal dentine^36^.

Further research should be carried out to test the mechanism behind the pigment extraction from the Juniper root, and its flow and accumulation in the skeletal/dental tissues. This is essential to substantiate the hypothesis described above. However, Cole and Waldron^37^ described a similar phenomenon in which skeletal remains exhibited a distinctive purple colouration. Authors mention the purple bone powder turned pink when placed in an acid solution, which could be comparable to stained bone in this study — the acidic impact of the soil, in which the remains were buried, could have contributed to the staining colouration. It is important to note that the colouration studied by Cole and Waldron^37^ seemed to evenly penetrate the full thickness of the bone, which is a differing factor to the present study. Nevertheless, the authors have also attributed the cause of the staining to likely be of a plant or fungal origin, potentially the *Aspergillus ficuum* species. Their research did not discuss the vegetation or other environmental factors present at the burial location; hence, the findings and further context contained in this study can be beneficial to attribute a more specific cause of this phenomenon.

Of additional relevance, the appearance of pink or purple discolouration on teeth in this specific assemblage and environment should be recognised as an environmental phenomenon and not mistaken for the postmortem ‘pink teeth’ phenomenon. In forensic literature, ‘pink teeth’ have been associated with asphyxial deaths such as strangulation or drowning; however, several studies indicate that the phenomenon is more commonly linked to haemolysis and decomposition rather than asphyxia^38^. In archaeological specimens, the pink discolouration likely has a different origin. For example, Dye et al.^39^ suggested it may result from postmortem changes caused by saprophytic fungi. The present research demonstrates that contact with trees of the conifer family can produce a similar colouration to that observed in ‘pink teeth,’ although, as shown, the aetiology of the two phenomena is entirely different.

Finally, the presence of entomological elements has provided crucial confirmation of information recorded over a century ago in a frontline diary describing events following a specific battle.

An important point to note before discussing the entomological finding is that S1 was buried with the skull facing east and positioned at the edge of the grave in a supine position. Similarly, S4 was buried supine, with the skull facing north at the edge and the feet pointing south. S6, in contrast, was interred on their right side, with the skull facing north toward the centre of the grave and the feet pointing south. Notably, the skulls of S1 and S6 were located at the periphery of the burial, and S1 and S6 were among the highest individuals in the grave, suggesting they were placed last, as mentioned in the Results section. All skeletal remains were found within the 0.70 to 0.80 m thick layer of the soil, consisting mostly of the filling deposit of the crater situated right below the superficial surface layer^3^.

Entomological findings from the analysis of S4 revealed the presence of the beetle *Pterostichus multipunctatus* inside the skull. This species is one of the most abundant ground beetles on Alpine grasslands; it is particularly abundant in June (the month in which the soldiers died, according to military diaries) and is a flightless species with very low dispersal ability^40^. Although this species is not typically associated with body infestation for nourishment, it is known to be particularly active in early summer, preying on dead animals (mainly invertebrates)^41,42^, but also deceased vertebrates^43–45^ and spends both its adult and larval stages on the ground surface, rarely burrowing deep into the soil^46^. This observation raises the possibility that the remains were either partially exposed or buried under only a thin layer of soil. Supporting this hypothesis, puparia of *Protophormia terraenovae* were found on S1, S4, and S6. This blowfly species has a wide Holarctic distribution and is particularly prevalent in colder regions, including the Arctic, where it has been recorded only as close as 850 km from the North Pole^47^. In the Alps, it has been collected at the beginning of summer from the carcass of a bear^48^, and it has also been reported in the context of the remains of World War I soldiers^6^. Like other members of the Calliphoridae family, *P. terraenovae* has been reported only colonising exposed bodies^45^. However, unlike those in the subfamilies Calliphorinae and Luciliinae, its larvae pupariate on or near the body from which they fed, further supporting the idea that the remains were at least partially exposed.

It is therefore plausible that the three individuals (S1, S4, S6), or at least their heads — since this is the only area where entomological elements were recovered — were either partially exposed or buried under a very thin layer of soil, which fits with the information regarding the depth of the grave at which the remains were located (mid-high to high in the grave column). This evidence indicates the accuracy and authenticity of a war diary that detailed the hasty burial of the fallen, interred in only a few centimetres of soil. Furthermore, it highlights the importance of collecting and studying all biological elements associated with human remains. The ability to confirm facts and testimonies from over a century ago underscores the potential value of such evidence, not only in reconstructing historical events but also in contributing to the validation of testimonies in contemporary investigations, such as cases of human rights violations or war crimes. In this sense, even a single point of corroboration may enhance the significance of a document and encourage investigators, whether judicial authorities or historians, to reconsider the relevance of fragmented or contested records. While this does not imply that such materials automatically qualify as credible primary sources, especially in an archaeological context, it does highlight their potential to serve as valuable informational assets within both historical and modern investigative contexts.

Overall, the collection of findings presented above is rarely addressed in taphonomic literature and can be valuable in forensic and bioarchaeological investigations. In this specific case and environment, the hypothesis that the preservation of skeletal elements decreases at lower depths due to SOM(Soil Organic Matter)-related acidic microenvironments within the grave is not supported by the statistical analysis. The lack of preservation is likely due to the complex taphonomic context of the mass grave, which includes generally low pH, plant root intrusion, leather contact, and localised water movement. The correlation between erosion and the direct contact of leather with skeletal elements requires further investigation. However, if this link is scientifically proven, it could contribute to more accurate interpretations of postmortem processes in archaeological contexts, particularly when personal items, biological and geological forces interact in historical mass burial settings. The imprint of specific plant species (or families) can be used to derive information about the taphonomic history in cases of secondary burials. While such plant-related staining can contribute to the reconstruction of taphonomic histories in any context, in forensic settings, secondary burials often result from illicit attempts to conceal a body. In such cases, the presence of specific botanical staining may provide investigative clues about the original deposition environment. This type of staining evidence could also be used in criminal cases in the same way as pollen and soil/sediment samples, to create an ‘environmental profile’ of the burial site^49^. In particular, the presence of reddish staining on teeth and skeletal elements buried away from conifers could potentially help identify areas where conifers are present in the primary burial area. This may be the first documented case of reddish phlobaphene staining on relatively recent human bone in an Alpine-meadow environment dominated by Juniperus, with well-drained soils. While not a definitive Alpine marker, it adds a potentially useful point of comparison in taphonomy and could aid in the interpretation of skeletal evidence discovered within this specific environmental context. Entomological evidence has validated century-old diary accounts of hasty burials following a battle, highlighting the importance of studying all the biological elements to corroborate investigations in both historical and contemporary contexts, such as human rights violations or war crimes.

## Conclusion

To conclude, this study offers important insights into how the complex interplay of taphonomic elements can enable a more accurate reconstruction of events associated with a mass grave, specifically, confirming a wartime episode that occurred a century ago. Broadening the perspective, it is important to highlight that in mountainous regions, taphonomic processes interact in intricate ways that challenge conventional models. The findings presented here have wide applicability across a range of extreme contexts, including mountaineering accidents – whether in the European Alps or other high-altitude regions worldwide – as well as political disappearances, military fatalities, and mass graves or unmarked burials of Indigenous peoples and persecuted minorities, as exemplified by exhumations carried out in the Peruvian Andes^50^. Ultimately, these results deepen the understanding of the taphonomic patterns in Alpine environments, underscoring their relevance for forensic science, archaeology, and related disciplines. They also provide a solid foundation for future research in these fields.

## Supplementary Information

Below is the link to the electronic supplementary material.

Supplementary Material 1

## Data Availability

The complete osteometric dataset and all derived data are available upon reasonable request. Because the skeletal remains and associated records are the exclusive property of the Italian State and classified as military heritage pursuant to D. Lgs. 42/2004 (Codice dei Beni Culturali e del Paesaggio), D.Lgs. 66/2010 art. 272 (Codice dell’Ordinamento Militare), and L. 365/1999, the release of the data requires prior written authorisation from the Ufficio Beni Archeologici of the Autonomous Province of Trento and the Ufficio per la Tutela della Cultura e della Memoria della Difesa (Ministero della Difesa). The human remains were reburied in accordance with the current Italian regulations governing military burials (e.g.: Decreto Legislativo 15 marzo 2010, n. 66). Therefore, no human remains of that mass grave are currently held in a museum or research repository. Personal and military items recovered during the excavation are stored and preserved at the Restoration Laboratory of the Archaeological Heritage Office of Trento Province. These materials are curated under the authority’s supervision and may be accessed for research purposes upon request.
