# Supplementary material for: Taphonomic patterns of a WWI Alpine mass grave: insights from the Italian front
Source: Sci Rep. 2025 Dec 22;15:45086. doi: 10.1038/s41598-025-32171-y (PMC12749954; doi:10.1038/s41598-025-32171-y)
Supplement: Supplementary file 1 — Supplementary Material 1 [file 41598_2025_32171_MOESM1_ESM.pdf]

Taphonomic Patterns of a WWI Alpine Mass Grave: Insights from the Italian Front.

Wiktoria Baranowska[1]*, Mauro Gobbi[2], Stefano Vanin[3], Franco Nicolis[4], Daniel Gaudio[1*]

Supplementary Information:

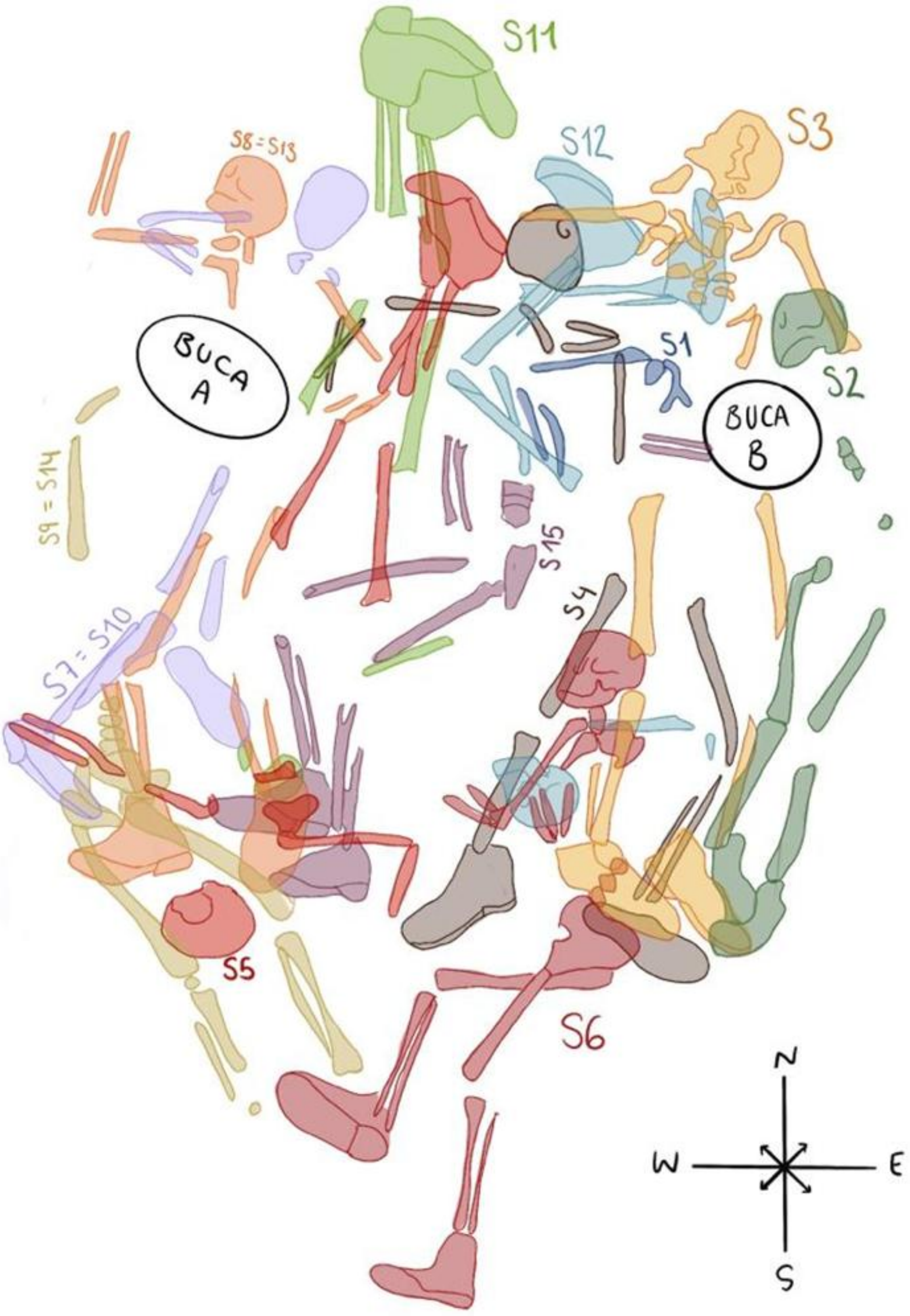

Supplementary Figure S1. *The diagram showing the approximate positions of the skeletal remains within the mass grave. The diagram was produced not to scale, based on the photographs from the excavation report produced by SAP[2]. Location of two holes illegally dug up by unknown individuals on the mass grave site were labelled as Buca A and Buca B.*

Test Statistics[a,b]

	API	QBI	F	F_PRESENT	BRI
Kruskal-Wallis H	1.092	1.045	1.487	1.780	.853
Df	3	3	3	3	3
Asymp. Sig.	.779	.790	.685	.619	.837

Supplementary Table S2*. Test statistics for Kruskal-Wallis test. a. Kruskal Wallis Test; b. Grouping Variable: Burial Depth.*

Types of bone staining present in skeletal assemblage from Cima Cady													
**Skeletal ID**	**S1**	**S2**	**S3**	**S4**	**S5**	**S6**	**S7= S10**	**S8= S13**	**S9= S14**	**S11**	**S12**	**S15**	**Potential causes[51]**
**Pink/ purple**	■	■	■	■	■	■	■	■	■	■	■	■	Potentially due to contact with Juniper roots penetrating the mass grave and bones.
**Green**		■	■						■	■	■	■	Likely associated with skeletal elements being in contact with military items made from copper or copper alloys.
**Orange**				■									Associated with direct contact with corroded iron personal items stuck to the skeletal element i.e. iron buttons.
**Dark staining of foot bones**		■	■	■	■	■	■	■		■	■	■	Likely due to leeching of tannins from boot leather.
**Black**						■							Likely due to direct contact with the gas mask recovered, that was in direct contact with the stained skeletal elements.
**Dark red**						■							Darker red staining than described in the paper, present on the interior of a single cranium – this has been hypothesised to either also come from contact with Juniper roots, or alternatively associated with the haemorrhage at the time of death of an individual (especially as present on the cranium also affected by blast/projectile trauma).
**Other**				■				■	■			■	White discolouration — bleaching of the bone most likely caused by UV radiation.<br>Raised grey-green patch — deemed to be a mixture of soil, roots and clothing fused onto the bone.

Supplementary Table S3. *Types of staining in the skeletal remains of Cima Cady mass grave. Blue colour is indicative of staining presence. Last column contains a brief explanation of attributed potential causes of the staining.*

INDIVIDUAL	SEX	AGE AT DEATH (YEARS)	STATURE (CM +/- STANDARD ERROR)	POPULATION AFFINITY	PATHOLOGIES	TRAUMA
**S1**	Probable Male	20-25	Not determinable	Not determinable	Caries, Signs of cribra orbitalia	-
**S2**	Probable Male	>22	164.6 ±3.37	European	Caries , calculus and dental abscesses, OA	High velocity projectile
**S3**	Male	21-30	Not determinable	Not determinable	Calculus, cribra orbitalia , signs of OA on the spine (osteophytes, Schmorl nodiles)	Blast /projectile trauma
**S4**	Probable Male	18-25	171.36 ±3.37	European	-	-
**S5**	Male	N/D	Not determinable	Not determinable	Caries and calculus	High velocity projectile trauma
**S6**	Probable Male	18-25	174.63 ±3.37	European	Calculus	Blast trauma/projectile trauma
**S7=S10**	Male	>20	Not determinable	European	Caries, calculus, antemortem loss of dental al elements (upper and lower molars)	-
**S8=S13**	Probable Male	>18	Not determinable	Not determinable	-	-
**S9=S14**	Probable Male	>18	164.70 ±3.27	Not determinable	OA of the spine and pelvis	High velocity projectile trauma
**S11**	Not determinable	>18	Not determinable	Not determinable	OA right tarsal and metatarsal	-
**S12**	Probable Male	>21	Not determinable	European	Calculus	-
**S15**	Probable Male	20-35	Not determinable	Not determinable	-	-

Supplementary Table S4. *Biological profile summary table fore the human remains recovered from Cima Cady.*
